# Abnormal functional responses of osteoblasts to leptin in adolescent idiopathic scoliosis

**DOI:** 10.1186/1748-7161-10-S1-O6

**Published:** 2015-01-19

**Authors:** Elisa MS Tam, Kar-Hing Yeung, Shengping Tang, Tsz-Ping Lam, Bobby KW Ng, Simon KM Lee, Yong Qiu, Jack CY Cheng

**Affiliations:** 1 Department of Orthopaedics and Traumatology, The Chinese University of Hong Kong, Hong Kong; 2 Oral Maxillofacial Surgery and Dental Unit, Prince of Wales Hospital, Hong Kong; 3 Department of Pediatric Orthopedics, Shenzhen Children's Hospital, Shenzhen, China; 4 Lee Hysan Clinical Research Laboratories, The Chinese University of Hong Kong, Hong Kong; 5 Spine Surgery, The Affiliated Drum Tower Hospital of Nanjing University Medical School, Nanjing, China; 6 Joint Scoliosis Research Center of the Chinese University of Hong Kong and Nanjing University, China

## Objective

Leptin has been postulated as one of the etiologic factors of AIS because of its important physiological functions in neuro-osseous development affecting skeletal growth, the onset of puberty, energy expenditure and body composition. Previous studies on the relationship between leptin and HR-pQCT derived bone quality parameters had found abnormal correlations in AIS girls, and suggested possible abnormalities in the leptin regulated bone metabolic pathways. Another study on AIS patients showed hyposensitivity to leptin in bone marrow derived mesenchymal stem cells This study aimed to investigate the effect of leptin on the functional responses of osteoblasts in AIS girls, and compare with that of controls.

## Material and methods

In vitro assays were performed with osteoblasts isolated from 12 severe AIS girls and 6 control subjects. The osteoblasts were exposed to different concentrations of leptin (0, 10, 100, 1000 ng/ml). The effects of leptin on cell proliferation were evaluated with MTT assay after 3 days of leptin treatment; differentiation with ALP activity assay after 6 and 14 days; and mineralization with von Kossa staining after 21 and 35 days.

## Results

In the control group, leptin significantly stimulated osteoblasts to proliferate with a dose dependent manner, while AIS group showed no proliferate response to leptin. Significant difference in MTT signal was detected between AIS and controls in 100 ng/ml leptin concentration. For differentiation, control group showed strong and significant trend in ALP activity to increasing leptin concentrations in both day 6 and 14, and no trend in ALP activity were observed in the AIS group. Significant difference in ALP activity was detected between AIS and controls in 100 ng/ml at day 6 and 1000 ng/ml leptin treatment at day 14. For mineralization, the control group showed mild increasing trend to increasing leptin concentrations, and again no trend was observed in the AIS group. Significant difference in the amount of calcium nodules was detected between AIS and controls in 1000 ng/ml leptin concentration at day 35.

## Conclusion

The results in this study suggested that the osteoblasts isolated from AIS girls had very low response to leptin when compared with controls. This decrease in response might due to dysfunction of leptin signaling pathway, which might include abnormalities in the leptin receptor or downstream signal molecules. This is an important finding and might serve to explain the low bone mass and deranged bone quality that is associated with AIS.

